# Improving *in vivo* release prediction from *in situ* forming depots with a novel flow-through *in vitro* dissolution apparatus^☆^

**DOI:** 10.1016/j.ijpharm.2025.125884

**Published:** 2025-08-20

**Authors:** Charlotte Peloso, Etienne Yvorra, Romain Delamare, Mélanie Campana, Sylvestre Grizot, Adolfo Lopez-Noriega

**Affiliations:** MedinCell S.A., 3 rue des Frères Lumi ère, 34830 Jacou, France

**Keywords:** Long-acting injectables, *In situ* forming depot, *In vivo* prediction, *In vitro* release, Flow-through equipment, *In vivo* mimicking environment

## Abstract

Having an *in vitro* dissolution method providing meaningful and translatable data is critical for early-stage research and development. For long-acting injectables, such as *in situ* forming depots (ISFD), the extended duration of the release test and the different release mechanisms involved do not allow the use of classical *in vitro* dissolution systems. To facilitate the transition from bench to animal studies, an alternative dissolution apparatus was developed. This flow-through system was designed to be more biorelevant, with the addition of temperature-controlled reaction chambers filled with an agarose hydrogel. This allows for a continuous constraint of the depot during dissolution testing as well as a continuous buffer flow in an open circuit. The release properties of the phase-inverting ISFD technology BEPO® were investigated with a large range of conditions (*i.e.* temperatures ranging from 25 °C to 45 °C and buffer flow rates from 1 to 10 mL/h) and different active pharmaceutical ingredients. The *in vitro* release profiles could be tailored while using the new dissolution apparatus. In particular, the temperature was the critical parameter, with higher temperatures leading to an increase of the release rate and generally more translatable *in vitro* release profiles to the respective *in vivo* data. Flow rate had a lower impact than temperature in modifying release kinetics. Overall, the most translatable release conditions were not the most biorelevant but led to comparable release profiles to those obtained *in vivo*. The *in vitro* setup developed can thus be considered an interesting and valuable surrogate to *in vivo* evaluation.

## Highlights

• Predicting *in vivo* release of *in situ* forming depots is challenging.
• A novel flow-through *in vitro* dissolution equipment was developed.
• *In vivo* predictability was improved with the flow-through apparatus.
• So far, *in vitro* release mechanisms are not biorelevant but release profiles are comparable to those *in vivo*.

## Graphical abstract

**Figure:**
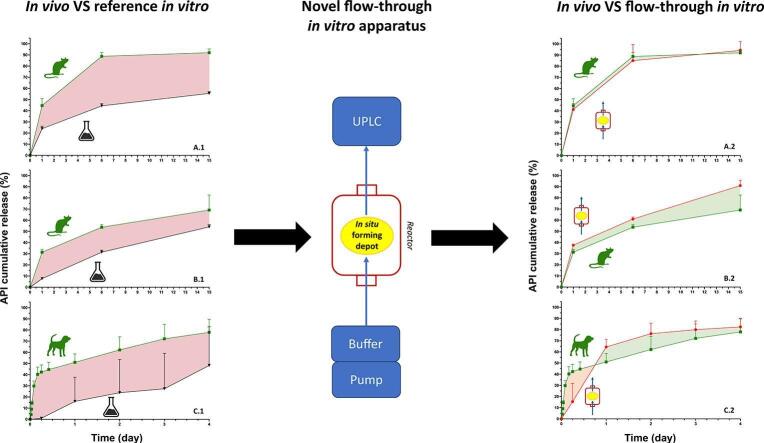

## 1 Introduction

The last decades have witnessed an extensive interest of the scientific community towards Long-Acting Injectables (LAI) (Hoffman, 2008, del Valle et al., 2009, Kleiner et al., 2014, Bauer et al., 2023, Alidori et al., 2024). Recognized for their improved efficacy and safety when compared to classic recurrent dosage treatments, they have the potential to significantly improve the patients quality of life by lowering the occurrence of side effects and potential intolerances, reducing the frequency of administrations and subsequently improving treatment compliance (Holm et al., 2023, Wright and Burgess, 2012, Wu et al., 2015, Owen and Rannard, 2016, Bartzokis et al., 2011, Kane et al., 2003, Gonella et al., 2022, Bauer et al., 2023). Moreover, these advantages optimize the therapies outcomes while reducing the overall healthcare costs (Kane et al., 2003, Pilon et al., 2017, Bauer et al., 2023), making them an interesting value-based proposition.

A variety of LAI strategies have been developed. One popular option is *in situ* forming depot (ISFD) technologies that allow a simpler and more patient-friendly administration procedure when compared to preformed implants (*i.e.* no microsurgery for the drug product administration and smaller injection needle size) (Kempe and Mader, 2012, Hatefi and Amsden, 2002, Gonella et al., 2022). Optimally, materials used for making ISFD should be bioresorbable to avoid surgical excision at the end of the treatment. However, the intricate release mechanisms of these systems pose significant challenges in characterizing their performance (Wright and Burgess, 2012, Gonella et al., 2022) and establishing *in vitro-in vivo* correlation (IVIVC), highlighting the need to develop advanced modeling and simulation techniques to improve *in vivo* predictability and *in vitro-in vivo* relationships (IVIVR) (Larsen et al., 2009, Bauer et al., 2023).

A large majority of ISFD products are administered by the parenteral route, with the subcutaneous route being preferred due to its ease of administration and minimally invasive nature (Wright and Burgess, 2012, Bauer et al., 2023). However, the United States Pharmacopeia (USP) dissolution methods were mostly developed focusing on oral delivery, omitting some physicochemical parameters specific to the parenteral routes of administration. Currently, the Food and Drug Administration (FDA) does not have a universally recommended dissolution method for testing *in vitro* release (IVR) of LAI, and more particularly ISFD products. The dissolution methods selected are presently dependent on the product to test (*i.e.* technology and/or active pharmaceutical ingredient (API)) (Bao et al., 2022, Iyer et al., 2006). To better suit the unique characteristics of ISFD, customizations of the systems can be made, generally with the USP Apparatus 4 (Flow-through Cell) (Shen and Burgess, 2012, Wright and Burgess, 2012, Iyer et al., 2007), as well as the USP Apparatus 2 (Paddle) and USP Apparatus 1 (Basket) (Burke and Koetting, 2021, Wang et al., 2023). Usually, research groups will favour setups derived from the sample and separate method because of their simplicity, with a release in a temperature-controlled buffer under a non-invasive shaking technique (*e.g.* orbital shaking instead of buffer stirring with paddle or magnetic bar), and sampling at regular intervals with or without buffer renewal (Rezaeian Shiadeh et al., 2023, Roberge et al., 2020, Hernandez et al., 2016, Leconet et al., 2018, Larsen et al., 2009). In all cases, particular attention must be given to the depot formation post-injection. Without physical constraints, the formulations can precipitate in random shapes, leading to various surface areas and subsequent release profiles (Wright and Burgess, 2012, Zhang and Fassihi, 2020). Control of the depot shape can be achieved by being pre-formed before the dissolution test (Zhang and Fassihi, 2020, Larsen et al., 2009), using molds (Suh et al., 2021, Shafiee et al., 2024, Kanwar and Sinha, 2019, Wang et al., 2024), or hydrogels to constrain the bolus during the release test (Ye et al., 2012, Hernandez et al., 2016, Leung et al., 2017, Gomes et al., 2024). Transition to *in silico* evaluation of depot release is also being investigated, with the development of complex computational models in an attempt to combine all the variables affecting a product’s performance *in vivo* (Shah and Hong, 2022, Corpstein and Li, 2023, Bannigan et al., 2023).

Depending on the API and specificities of the Targeted Product Profile (TPP), a large spectrum of *in vitro* release profiles can be obtained. However, the evaluation of the same formulations *in vivo* does not systematically highlight the same discrimination observed *in vitro*, showing the potential false and misleading discrepancies obtained with current *in vitro* setups. The need for a more robust and reliable IVR setup is critical to improve the *in vitro* development of ISFD drug products and improve their selection for preclinical studies, speeding up the full development phase before clinical trials. Ideally, this IVR test should accurately mimic the physiological environment of the targeted *in vivo* injection site, including biological, physicochemical and mechanical elements (Patel et al., 2010, Larsen et al., 2009).

In this article, the LAI technology based on an *in situ* forming depot phase-inverting system, registered as BEPO® (Gaudriault 2011), was investigated. This commercial-stage bioresorbable technology is based on the precipitation of a PEG-PLA copolymer mixture once in contact with biological fluids. An organic solvent will be released from the preparation, leaving a semi-solid copolymer shell entrapping a pharmaceutical ingredient and ensuring its progressive release from days to months (Roberge et al., 2020, Leconet et al., 2018, Molinier et al., 2021). Commonly, IVR from these formulations are monitored using a direct dissolution method with a gelatin capsule as a temporary mold (Suh et al., 2021, Roberge et al., 2020). This method allows the formulation to be contained while it undergoes phase exchange, leading to precipitated depots of reproducible morphologies and generally reducing the variability of results. However, the resulting *in vitro* release profiles are occasionally misleading for the selection of preclinical candidates, resulting in some unexpected *in vivo* release profiles.

To minimize this uncertainty, an alternative dissolution apparatus for *in situ* forming depot technologies has been developed by MedinCell S.A. (Jacou, France). The custom-made apparatus presented in this article incorporates certain aspects of parenteral administrations that are generally omitted in classic dissolution setups, aiming to improve the translatability of *in vitro* to *in vivo* results. The *in vitro* release of meloxicam and bupivacaine BEPO® formulations was evaluated using this apparatus and compared to a direct dissolution method and *in vivo* release in animal models. These initial IVR tests allowed the identification of the apparatus’ optimal operating parameters to better anticipate *in vivo* performance of the formulation. Eventually, the relevance of the setup was challenged with one formulation of 4-ethynyl-2-fluoro-2-deoxyadenosine (EFdA), a novel antiretroviral, tested both *in vitro* and in a pharmacokinetic study.

## 2 Materials and methods

### 2.1 Materials

Diblock (DB) and triblock (TB) copolymers based of polyethylene glycol (PEG) and polylactic acid (PLA) were produced by CM Biomaterials (Tucker, GA, USA). Two triblocks (*i.e.* TB1 and TB2 with TB1 of a higher molecular weight than TB2) and two diblocks (*i.e.* DB1 and DB2 with DB1 of a higher molecular weight than DB2) were used in this study. The organic solvent used in this study was Dimethyl Sulfoxide (DMSO) USP grade (Procipient®) from Gaylord Chemical (Los Angeles, CA, USA). Phosphate-buffered Saline (PBS) solution (pH 7.4) was prepared by dilution of a PBS 10X solution (BP399-20, Fisher Bioreagents, Waltham, MA, USA) in ultrapure water. Meloxicam was supplied by Swati Spentose (Mumbai, India), bupivacaine by Interchim (Montluçon, France), and 4′-Ethynyl-2-fluoro-2′-deoxyadenosine (EFdA) by BioDuro (Beijing, China).

### 2.2 Formulation preparation

Polymeric BEPO® vehicles were prepared by mixing the appropriate amount of TB, DB and DMSO in a glass vial on a roller mixer at 40 rpm at room temperature (RT) until complete visual dissolution. The obtained solutions were clear, translucent and viscous. The appropriate amount of active pharmaceutical ingredient (API) was added to the vehicle and left again on a roller mixer at RT until solubilization is complete. All prepared formulations were solutions. Details of the formulation compositions are presented in Table 1.

**Table 1:** Formulations compositions.

API	% wt.	TB:DB ratio	% wt.	Solvent	% wt.
Meloxicam	1.5	TB1:DB1 1:1	40	DMSO	58.5
Bupivacaine	5.0				55.0
4′-Ethynyl-2-fluoro-2′-deoxyadenosine	2.5	TB2:DB2 1:1			57.5

API: Active Pharmaceutical Ingredient; wt.: weight; TB: triblock; DB: diblock; DMSO: Dimethyl Sulfoxide.

### 2.3 Analytical equipment and methods

API was quantified by Ultra-Performance Liquid Chromatography (UPLC) on an ACQUITY UPLC H-Class PLUS system (Waters Corporation, Milford, MA, USA). DMSO was quantified by High-Performance Liquid Chromatography (HPLC) on a 1260 Infinity II LC system (Agilent Technologies, Santa Clara, CA, USA). Details on the analytical methods used are presented in Table S1. Chromatographic analysis and API quantification were performed using the software Empower™ (Waters Corporation, Milford, MA, USA) version 3.8.0.1.

Release rate (mg/day) was calculated from the cumulative API quantified at each interval according to Eq. (1).

(1) $$m_{APIbetweent1andt2}/\left(t2-t1\right)$$

### 2.4 API stability and solubility in PBS

Experiments were conducted at room temperature (RT) (*ca.* 25 °C), 37 °C and 45 °C. API stability in PBS (release medium) was assessed as well as its solubility. Briefly, a minimum of 5 mg of API were weighed in a 5 mL glass vial, and 2 mL of buffer (*i.e.* either at RT, 37 °C or 45 °C) were added on top of the powder. The vial was closed and vortexed for 30 sec. If a solution was obtained, more API was added to the preparation and re-vortexed. If a suspension was visually obtained, the vial was stored in the appropriate temperature condition under orbital shaking (74 rpm). After 24 h and filtration through a 0.2 µm hydrophilic polytetrafluoroethylene (PTFE) membrane, the API concentration in the obtained solution was determined by UPLC analysis. The measured concentration was considered as the solubility value of the API at each temperature. The UPLC chromatograms were also compared to API standards to detect any traces of degradation or alteration to the API structure.

### 2.5 *In vivo* studies

*In vivo* studies were conducted by Porsolt (Le Genest-Saint-Isle, France) and Avogadro LS (Fontenilles, France), in accordance with the Directive 2010/63/EU for the protection of animals used for scientific purposes. Each facility was accredited by the Association for Assessment and Accreditation of Laboratory Animal Care (AAALAC).

#### 2.5.1 Ex vivo API release dosage study with depot extraction

The release kinetics of formulations of meloxicam and of bupivacaine were assessed during a 15-day *in vivo* study in male Wistar rats (studies authorization numbers 2,019,020,512,422,975 and 2,017,022,811,072,768 respectively). Approximately 200 mg of formulation (corresponding to a volume of 170 µL) was injected subcutaneously into the flank of nine animals. Three rats were euthanized per time point (*i.e.* 1, 6 and 15 days). Depots were recovered and remaining API and DMSO were quantified *ex vivo* (n = 3 for meloxicam formulation, n = 2 for bupivacaine formulation due to other characterizations needed for this test item). This protocol allowed to determine the experimental *in vivo* API release at a specific timepoint while bypassing the absorption, distribution, metabolism and excretion (ADME) processes.

#### 2.5.2 Classic pharmacokinetic study and data analysis

EFdA formulation release kinetics were assessed in an 84-day pharmacokinetic (PK) study in male Beagle dogs (study authorization number 2017022811072768). A dose of 672 mg of formulation (equivalent to 580 µL) was injected subcutaneously in the interscapular area of four animals. Blood samples were taken regularly in vacuum tubes containing K2-EDTA as an anticoagulant agent for plasma preparation. Samples were centrifuged at 2700 rpm for 10 min at 4 °C and stored at −80 °C until bioanalysis at the University of North Carolina (Chapel Hill, USA). After 84 days, the study was stopped, the animals euthanized, and the injection sites recovered for depot extraction. The remaining API content in the depot was determined. No DMSO dosage was performed. Deconvolution was performed using Phoenix WinNonlin™ (Certara, Pennsylvania, USA) version 8.5 to evaluate *in vivo* cumulative drug release and delivery. The methodology is based on linear system analysis with linearity defined in the general sense of the linear superposition principle. The concentration of a drug function, C(t), is defined by Eq. (2).

(2) $$C\left(t\right)=f\left(t\right)\times ⨂\times c_{d}\left(t\right)$$

where ⨂ is used to denote the convolution operator and c_d_(t) is the unit impulse response (UIR), also known as the disposition function. Eq. (2) is the key convolution equation that forms the basis for the evaluation of the drug input rate, f(t). Phoenix WinNonlin™ deconvolution uses the basic principle of deconvolution through convolution (DTC) to determine the input function. The DTC method is an iterative procedure consisting of three steps:

1) the input function is adjusted by changing its parameter values.
2) the new input function is convolved with c_d_(t) to produce a calculated drug level response.
3) the agreement between the observed data and the calculated drug level data is quantitatively evaluated according to some objective function.

The three steps are repeated until the objective function is optimized.

The Deconvolution assumes a UIR function of the form of Eq. (3).

(3) $$c_{d}\left(t\right)=\sum_{j=1}^{N}A_{j}e^{-ajt}$$

where A is the coefficient for a dose unity (with concentration unit), α is the exponent for disposition (with 1/time unit), and N is the number of exponential terms. To estimate the drug input rate f(t), the mean UIR parameters A_j_ and α_j_ were estimated from concentration-time data following instantaneous input (IV infusion) of EFdA using PK modelling. A 2-compartment model was used to describe the EFdA plasma concentration after an IV infusion to dogs.

## 3 Reference *in vitro* release setup with gelatin capsules

The reference IVR setup was an adaptation of a sample and separate method. IVR studies were performed in triplicate. The body of a gelatin capsule (Coni-Snap®, Capsugel, Morristown, NJ, USA) was stabilized upright on a scale. Capsules size 1 and size 000 were used for 170 µL and 580 µL of formulation, respectively. The appropriate formulation amount was added inside the unclosed capsule, which was then immersed in a 250 mL Erlenmeyer flask containing 100 mL of PBS buffer (pH 7.4) at 37 °C. The Erlenmeyer flasks were stored in a climatic room at 37 °C under continuous orbital shaking (74 rpm). The gelatin capsule dissolved within the first 30 min of the test, letting free a precipitated depot with an outer shell and eventually a full precipitation thereafter. At given time points, the medium was sampled using a 2.5 mL Luer Lock syringe (Terumo, Tokyo, Japan) and filtered through a Phenex™ 0.2 µm RC membrane syringe filter (Phenomenex, Torrance, CA, USA) for API and DMSO dosage by liquid chromatography (LC). The total buffer content was subsequently discarded and replaced with 100 mL of fresh pre-heated buffer. At the end of the study, the buffer was sampled, discarded and the depot was dissolved in 4 mL of acetonitrile. This solution was diluted either in a mixture of acetonitrile and water for API dosage, or pure water for DMSO dosage.

### 3.1 Description of the flow-through *in vitro* release system

**Fig. 1:**
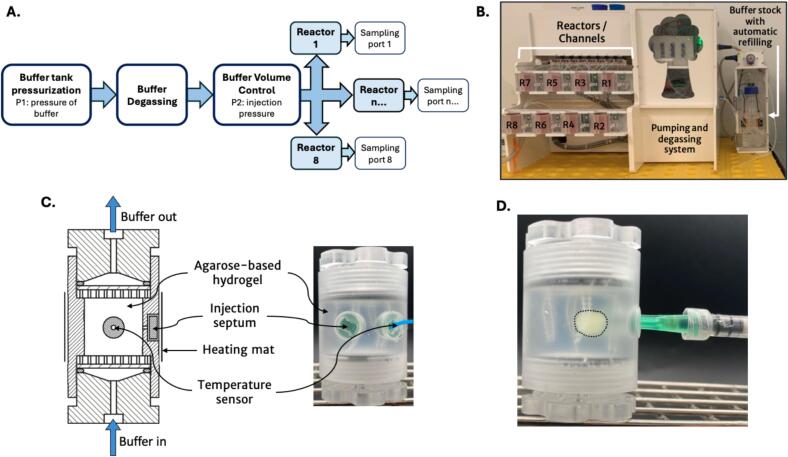
Presentation of the flow-through IVR apparatus. A. Functional block diagram of the equipment, B. General view of the equipment, C. Reactor description, D. Picture of a 200 mg depot in a reactor immediately post-injection.

The system is an innovative flow-through dissolution equipment designed for analytical precision and efficiency (Fig. 1). It is composed of a pressurized bottle that serves as the reservoir for the PBS buffer, currently used to simulate basic physiological conditions. A custom-built degassing unit is incorporated at the reservoir outlet to prevent bubble formation within the reactors. This unit includes a porous capillary enclosed in a vacuum chamber, effectively removing dissolved gases from the liquid and enhancing experimental reproducibility. The degassed buffer is distributed into multiple reactors through PTFE tubing (750 µm diameter). The flow of buffer through this system is tightly regulated by a home-made Volume Flow Controller (VFC). The VFC can maintain flow rates ranging from 1 to 10 mL/h and can spread a maximum pressure of 500 mbar. This precise control is vital for overcoming fluidic resistance and ensuring a consistent flow rate across all reactors.

Each reactor in the system is custom-designed and constructed from polymethylmethacrylate (PMMA), known for its robustness and chemical resistance, making it ideal for this application (Fig. 1C and D). The reactors are dimensionally tailored with an internal cylinder chamber of 17 mm in height for a radius of 20 mm. Reactors are equipped with individual heating pads and temperature probes, which allow for the precise control of the reactor temperatures in a range from 25 °C (RT) to 55 °C. Reactors contain a specific hole filled with a PTFE septum to allow direct injection into the device under a buffer flow. Each apparatus can accommodate up to 8 reactors (Fig. 1B).

In this study, the reactors were filled with a hydrogel, which was prepared by mixing 0.2 % weight agarose powder (Sigma-Aldrich, Saint-Louis, MO, USA) into a PBS solution. This agarose concentration was selected to ensure hydrodynamic permeability. The preparation was heated to 90 °C for 5 min and left to cool 10 min at room temperature. Flexible polymer scaffolds were incorporated into the reactors before the agarose mixture was poured. These polymer filaments are positioned inside the reactors and incorporated in the agarose hydrogel to ensure its mechanical stability and prevent its disassembly or collapse. The hydrogel will use this extra surface area to resist the laminar buffer flow going through its pores. The liquid agarose mixture was left into the reactors at RT for 2 h to ensure complete gelation. Finally, the reactors were closed on both sides with a 0.2 µm nylon membrane (0.45 µm porosity) and connectors to the tubing. Buffer was injected at both reactor entry and exit to avoid trapping air within the system. The reactors were connected on the flow-through apparatus and equilibrated under a constant flow rate.

### 3.2 *In vitro* release testing in the flow-through apparatus

The hydrogel-filled reactors were put at the appropriate temperature and buffer flow rate conditions for at least 30 min before injection. For testing at 25 °C (RT), no setpoint was specified in the heating system (*i.e.* left at room temperature) while testing with temperatures above 25 °C were controlled by the heating system from the flow-through apparatus. For the screening sets, 3 time points were evaluated with 2 replicates launched per time point. For the validation sets, 4 replicates were followed per condition. The tested formulation was prefilled in an Omnifix®-F Luer Lock Solo 1 mL syringe (B.Braun, Melsungen, Germany) equipped with 21G 5/8″ Agani^TM^ needle (Terumo, Tokyo, Japan), which was weighed before injection. The 21G 5/8″ needle was inserted through the reactor septum to reach the middle of the hydrogel. The formulation was injected at a continuous speed (about 3 mL/min) without moving the needle, reducing the risks of widely disrupting the hydrogel along the needle path. The needle was kept in place 5 s post-injection, allowing the depot to start precipitating and facilitating the depot detachment from the needle tip. After injection, the exit tubing of the channel was placed in a flask to collect the buffer flow-through. The flasks were collected at regular intervals (*i.e.* daily to every 3 days, allowing to neglect buffer vaporization), replaced by empty ones, and the collected buffer volume was measured to verify the experimental flow rate. The collected buffer was subsequently sampled for API and DMSO dosage. At study termination, the flow rate was stopped and the reactors disconnected. The depots were isolated from the hydrogels and treated as described in Section 2.4 for API and DMSO quantification.

### 3.3 Polymeric depot characterization

Depots were macroscopically evaluated at given time points during the reference IVR studies and at termination of the studies from both the reference setup and the flow-through apparatus. The general depot shape and texture were qualitatively assessed. The depots were also weighed on a precision scale to evaluate their water uptake during the study. The depot weight initially injected was corrected by the amount of released DMSO and API at the measured time point. The copolymers selected were known to not degrade over the tested duration (*i.e.* maximum of 15 days). The water content at time (t) was calculated with Eq. (4) and the water uptake (WU) at time (t) with Eq. (5).

(4) $$m_{\mathrm{water}}\left(t\right)=m_{\mathrm{depot}}\left(t\right)-m_{\mathrm{injected}}-m_{\mathrm{DMSO}+APIrelaesed}\left(t\right)$$

(5) $$\mathrm{WU}\left(t\right)=\frac{m_{\mathrm{water}}\left(t\right)}{m_{\mathrm{injected}}}\times 100$$

### 3.4 Statistical analysis

Data are reported as means with standard deviation (SD). The relative standard deviation (RSD) was calculated at each IVR time point for each setup to assess the variability of the methods.

## 4 Results

### 4.1 Predictability level of the reference IVR setup

**Fig. 2:**
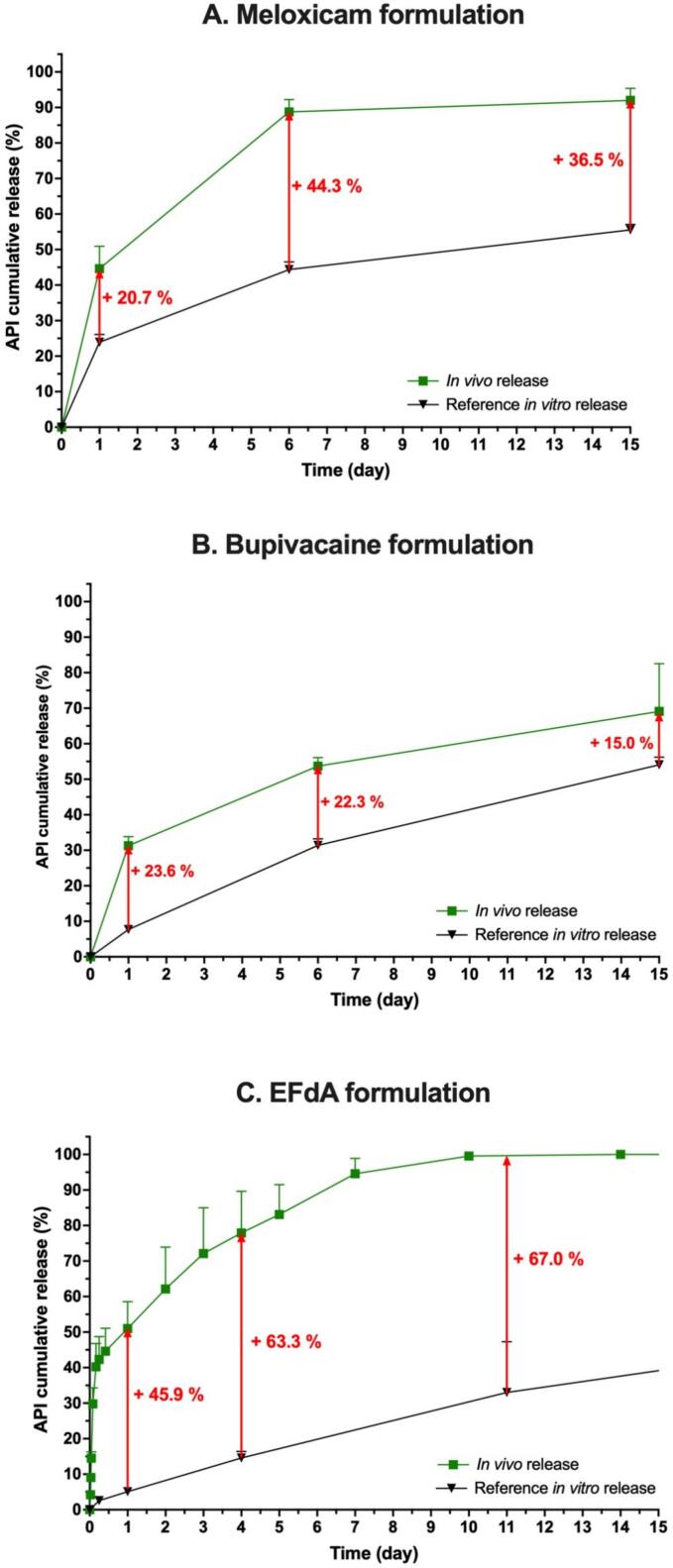
Cumulative API release profiles comparison between *in vivo* and *in vitro* data using a reference IVR setup in PBS. A. Meloxicam formulation, B. Bupivacaine formulation, C. EFdA formulation.

The *in vivo* release kinetics of model formulations of meloxicam, bupivacaine and EFdA were compared to their corresponding IVR using a reference setup (*i.e.* sample and separate method as described in section 2.4). At first, the IVIVR was investigated. For meloxicam and bupivacaine formulations, *in vivo* release profile was obtained after depot extraction and dosage of remaining API in the explants at 1, 6 and 15 days. The amount of non-released drug was used to determine the percentage of released cargo and a cumulative release curve was established. For EFdA formulation, API was quantified in plasma during a classic pharmacokinetic (PK) study with multiple blood samplings. The plasma concentration over time was plotted and remaining API contents in the depots were quantified at study termination (84 days). A deconvolution method was used to convert the results into an *in vivo* cumulative release profile. The plasmatic profile and resulting cumulative release profile are presented in Fig. S1. Fig. 2 highlights the disparity between *in vitro* and *in vivo* profiles for these three formulations.

At Day 1, the amount of API released from the depots already differed by more than 20 % between *in vitro* and *in vivo* testing. For meloxicam formulation, the discrepancy became even greater at Day 6 while it remained relatively constant for bupivacaine formulation.

In the case of EFdA formulation, *in vitro* release lasted over 2 months. However, the API was only quantifiable over 2 weeks after injection in dogs, with a substantial initial burst (*i.e.* about 50 % in 1 day). The *in vitro*/*in vivo* difference ranged from +45.9 % at Day 1 to +57.8 % after Day 17, with no obvious adjustment of the *in vitro* release to match the *in vivo* profile. In all cases, the initial burst was always lower *in vitro*, which poorly represented the preclinical data.

### 4.2 Relevance of the flow-through apparatus compared to the reference IVR setup

The performance of the flow-through apparatus was initially evaluated using the meloxicam and the bupivacaine formulations. The influence of temperature on API release was tested first. IVR were followed over 15 days at room temperature, equivalent to *ca.* 25° (*i.e.* no heating) and 45 °C. No impurities were detected by LC after 15 days of incubation at 45 °C, suggesting stability of both APIs (Fig. S2). The flow rate was initially set at 1 mL/h and remaining API quantity was determined within the depots after 1, 6 and 15 days of incubation, to be comparable to the data generated in the corresponding *in vivo* study.

**Fig. 3:**
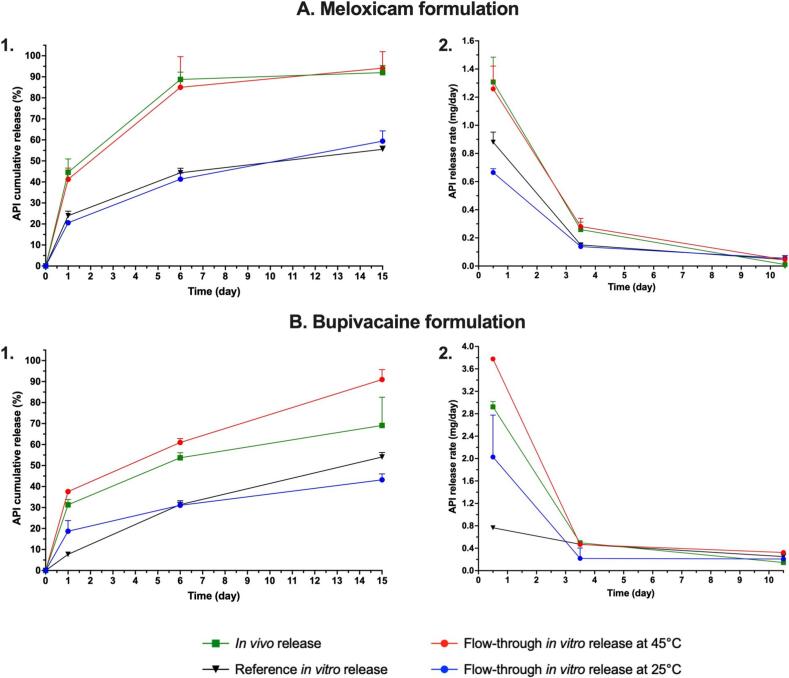
Comparison of mean API release profiles and standard deviation with different release setups over 15 days. A. Meloxicam formulation. B. Bupivacaine formulation. 1. Cumulative release percentages. 2. Release rates in mg/day at the midpoints of each interval. IVR were all performed in a PBS medium.

A higher temperature led to an increase of the API release in the flow-through apparatus for both meloxicam (Fig. 3A) and bupivacaine formulations (Fig. 3B). Interestingly, the release profile at 25 °C in the flow-through apparatus was comparable to the one obtained with the reference IVR setup at 37 °C, whereas the profile at 45 °C in the flow-through apparatus was very close to the *in vivo* profile. It is noteworthy that the main difference between the profiles came from the API burst at Day 1, as highlighted by the release rate representation in Fig. 2C and D. After Day 6, the release rate was similar for all the conditions and setups. In terms of method variability, the RSD between replicates was always below 20 % (Fig. S3 A. and B.) and was systematically below 10 % for the reference *in vitro* setup (*i.e.* mean RSD of 5.6 % and 4.1 % for meloxicam and bupivacaine formulations respectively), highlighting a good reproducibility of the method for these formulations. Regarding the flow-through experiments, RSD was below 10 % for testing at 25 °C of meloxicam formulation (*i.e.* mean RSD of 5.2 %) and 45 °C of bupivacaine formulation (*i.e.* mean RSD of 3.1 %). For the flow-through testing at 45 °C of meloxicam, the mean RSD was observed at 12.8 % while it was at 12.7 % for the bupivacaine formulation at 25 °C, highlighting a lower reproducibility of these methods, especially at early time points.

**Fig. 4:**
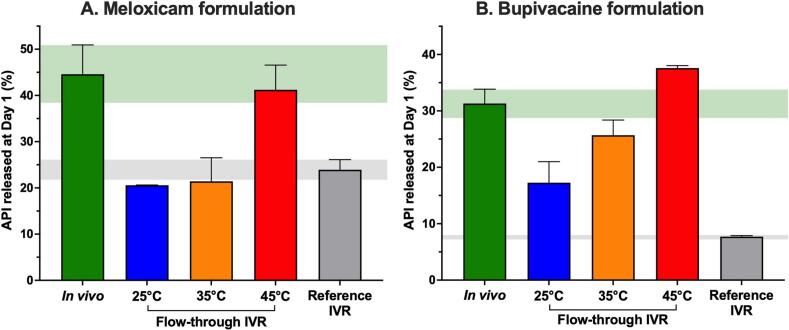
Comparison of mean API releases from meloxicam (A) and bupivacaine (B) formulations after 1 day at different flow-through IVR temperatures. Flow rate was fixed at 1 mL/h. IVR were all performed in a PBS medium.

In addition to this initial testing, the same temperature conditions were investigated in the flow-through apparatus with a higher flow rate, *i.e.* 10 mL/h. As the burst level at Day 1 had previously been observed as the critical readout, conditions were investigated over one day. Overall, the flow rate did not significantly modify the API burst, and the temperature was confirmed as the main factor modifying the release (Fig. S4). An arbitrary intermediate temperature was also evaluated in the flow-through equipment (*i.e.* 35 ± 5 °C) under a 1 mL/h buffer flow rate (Fig. 4) with n = 4 replicates.

Once again, changing the temperature in the flow-through reactors modified the burst magnitude and confirmed that temperature is the critical parameter to tailor the release in the flow-through equipment. The burst value at 35 °C was between those observed at 25 °C and 45 °C. Overall, the release at 35 °C in the flow-through apparatus was alternatively similar or higher than the capsule IVR at Day 1, leaving the 45 °C flow-through condition as the most comparable to the *in vivo* data in rats. Variability at 35 °C was similar to other temperatures in the flow-through apparatus (*i.e.* RSD of 11.2 % and 10.4 % for meloxicam and bupivacaine formulations respectively).

### 4.3 Exploration of the release mechanisms

As a temperature increase could lead to differences in API solubility within the release buffer, the meloxicam and bupivacaine solubilities in PBS were measured at all tested temperatures (Table 2). For meloxicam, a steady increase of the API solubility was observed with the increase of the temperature. The solubility at RT was increased by a 1.5- and 1.7-fold at 37 °C and 45 °C respectively. On the other hand, bupivacaine solubility slightly decreased with the increase of temperature.

**Table 2:** API solubility in µg/mL (standard deviation) at 24 h in PBS at different temperatures with n = 3 measurements.

	RT (25 °C)	37 °C	45 °C
**Meloxicam**	515 (1)	779 (1)	897 (2)
**Bupivacaine**	320 (1)	292 (0)	275 (2)

**Fig. 5:**
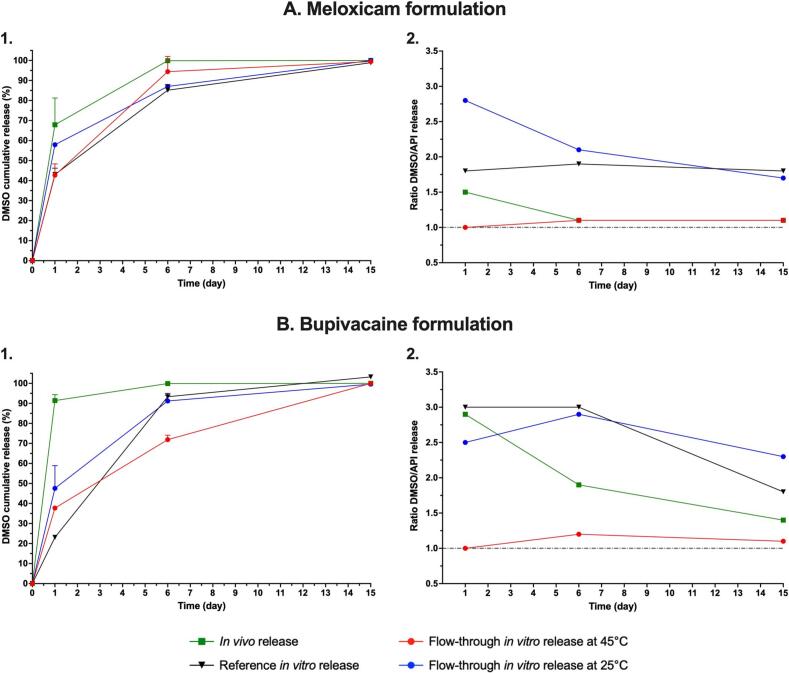
Comparison of the DMSO release from meloxicam (A) and bupivacaine formulation (B). 1. DMSO cumulative percentage releases in different *in vitro* setups. 2. Ratio DMSO/API percentages release during the study.

It was previously showed how the API release could be linked to the phase exchange phenomenon of depot formation and the DMSO release kinetics (Roberge et al. 2020). Therefore, DMSO was also quantified in the depots at each time point. Cumulative percentage releases of DMSO over API are compared in Fig. 5.

In all cases, DMSO release was complete after 15 days, with the ranking between the IVR setups varying between the two model formulations (Fig. 5A and C). Similarly to the API, the *in vivo* burst phase (*e.g.* Day 1) was the most critical and difficult portion of the release to simulate *in vitro*. Of all the conditions, the DMSO release with the flow-through apparatus at 25 °C tended to be the closest to the release observed *in vivo*. DMSO release after one day with the flow-through apparatus at 45 °C was always lower than the one at 25 °C, and similar or slightly above the reference IVR setup.

Fig. 5B and D highlights the substantial DMSO/API ratio variation depending on the setups. *In vivo*, the DMSO burst at Day 1 was higher than the API burst (*e.g.* ratio at 1.5 and 2.9 for meloxicam and bupivacaine formulations respectively). Nonetheless, the ratio decreased overtime to get closer to 1 after 15 days, with almost no DMSO retrieved in the depots at Day 6. With the reference IVR setup, the ratio at Day 1 is similar to *in vivo* (*i.e.* 1.8 for meloxicam and 3.0 for bupivacaine formulations). However, the measured quantity of DMSO released is 24.9 % and 68.3 % lower for meloxicam and bupivacaine formulations respectively, when compared to the *in vivo* data. With the flow-through setup at 25 °C, the ratio is variable, always over 1.7, and tends to be similar to the reference IVR setup from Day 6. Finally, the DMSO cumulative release percentage in the flow-through apparatus at 45 °C is systematically superimposable to that of the API (*e.g.* ratio between 1.0 and 1.2), independent of the formulation. Moreover, this DMSO release was comparable to that of a reference IVR setup, so lower than the one observed *in vivo*.

**Fig. 6:**
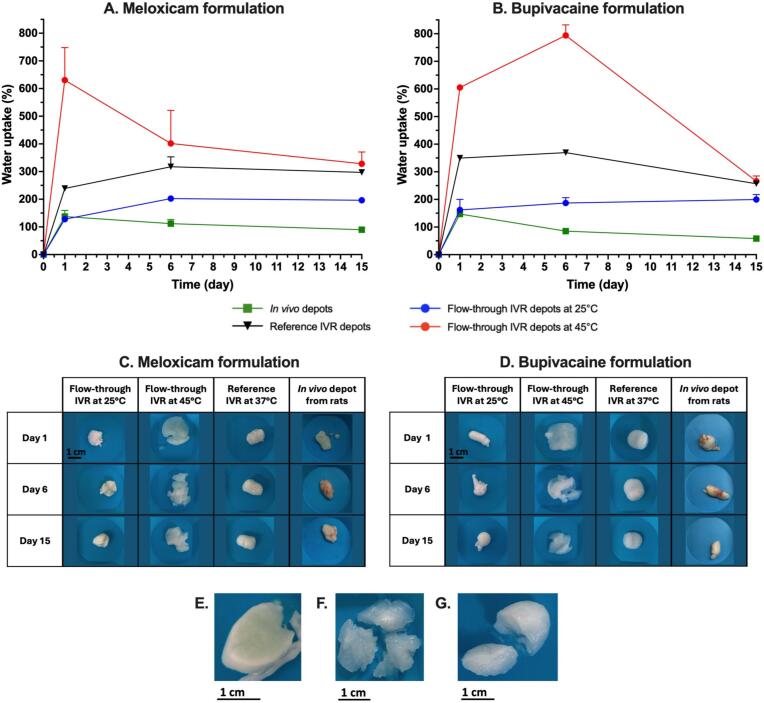
Mechanical behaviour of meloxicam (A and C) and bupivacaine (B and D) formulations depots in different release setups. A and B present the water uptake of the formulations over the course of the study. C and D present the morphology of the depots in each condition. Images E to G are representative cuts of depots formed (E) in a flow-through setup at 25 °C or (F) 45 °C, or (G) with a reference IVR setup.

Retrieved explants from the *in vivo* study and depots collected from the different IVR setups at each time point were macroscopically observed and weighed to determine their water uptake (Fig. 6).

Depots at 45 °C in the flow-through apparatus underwent a large swelling and accumulated buffer, with a water uptake 4.6- and 4.1-fold more pronounced for meloxicam and bupivacaine formulations respectively, compared to the *in vivo* depots after one day. In the reference IVR setup, the water uptake was relatively stable from Day 1 (*e.g.* 1.7- and 2.4-fold larger for meloxicam and bupivacaine formulations respectively) and remained higher than *in vivo*. Finally, depots at 25 °C in the flow-through apparatus presented a water uptake similar to *in vivo* at Day 1, with a stable water uptake for the rest of the study instead of a progressive decrease characteristic of *in vivo* depots.

From a macroscopic point of view, the depots formed at 25 °C tended to form compact spherical or ellipsoidal depots. When cut, a strong white outer layer was observed with a structured core (Fig. 6E). These structures were also observed in the depots recovered from the *in vivo* study (visual observation, but no pictures were taken). On the other hand, depots formed at 45 °C swelled and expanded within the hydrogel. The depots were spheroidal or ellipsoidal but very fragile with a looser and less organized structure than at 25 °C (Fig. 6F). Also, they were visibly filled with buffer. Finally, depots formed in the reference IVR setup were semi-solid, with an ellipsoidal shape conferred by the gelatin capsules. Depots swelled more than those at 25 °C and presented a relatively firm, flawless texture, intermediate between the strong and soft depots observed at 25 °C and 45 °C respectively (Fig. 6G). These observations were comparable for both formulations. The depot aspects were similar from Day 1 until the end of the study, with only the degree of swelling evolving over time.

### 4.4 Validation of the system with a classic pharmacokinetic study

To confirm the first observations made on model formulations, *in vitro* testing was conducted in the flow-through equipment using a candidate formulation tested in a classic pharmacokinetic (PK) setup. Formulation containing EFdA, a relatively soluble molecule, was selected for its substantial disparity between *in vivo* and *in vitro* release profiles when using the reference *in vitro* setup. The *in vivo* study was conducted in Beagle dogs, allowing the injection of a larger depot volume (*i.e.* 672 mg of formulation, equivalent to a 580 µL injection volume). Blood sampling was performed at defined time points to generate a drug plasma concentration profile, which was transposed into a cumulative release profile thanks to a deconvolution method. To mimic a similar dosing procedure, the buffer flowing through the reactors was collected and analysed by LC to generate the API cumulated release.

The impact of the flow rate on this new API was re-evaluated over 1 day with an additional flow rate of 5 mL/h. This flow rate was added in the screening to have an appropriate buffer volume to collect at each sampling interval, without an extensive need to dilute the samples for subsequent LC dosage.

**Fig. 7:**
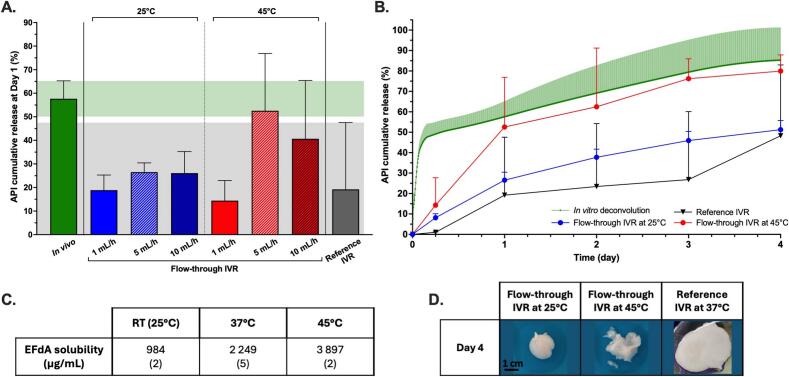
EFdA formulation data set. A. Comparison of the API release at Day 1 with different PBS flow rates in the flow-through equipment. B. API release in different setups, with a 5 mL/h PBS flow rate for the flow-through IVR setup. C. Solubility values (standard deviation) of EFdA after 1 day of storage in PBS at different temperatures (n = 3 measurements). D. Depot morphology after 4 days in the different IVR setups.

Fig. 7 A confirms that the flow rate had no impact on the API release at 25 °C. At 45 °C, the intra-condition variability was very high, meaning there was no major difference between the flow rate conditions (*i.e.* RSD of 59.4 %, 46.3 % and 60.78 % for 1, 5 and 10 mL/h respectively). However, a trend could be noticed for a larger burst at Day 1 for higher flow rates. As the 5 mL/h flow was considered more appropriate in terms of buffer consumption and API dilution, this flow rate was selected for testing over 4 days.

The release profiles in all tested conditions are compiled in Fig. 7B. The flow-through IVR at 45 °C led to the most predictive *in vitro* release profile once again, even if its initial API burst at 6  h was lower than that observed *in vivo*. However, the general profile matched the PK data from Day 1 to Day 4. Again, the release obtained at 25 °C in the flow-through apparatus was slower than that at 45 °C, but quicker than the API release kinetics generated with the reference IVR setup. Stability of EFdA was also confirmed over 4 days as the whole API cargo was recovered from the flow-through and the remaining depot quantification. In this case study, EFdA solubility in PBS (Fig. 7C) increased with temperature (*i.e.* 1.7 and 4-fold higher at 37 °C and 45 °C respectively). Globally, using the flow-through apparatus for IVR testing allowed to reduce the *in vitro* variability previously observed with the reference IVR method, by reducing the mean RSD obtained over 4 days from 119 % with the reference IVR, to 11 % at 25 °C and even 29 % at 45 °C in the flow-through equipment (Fig. S3 C.).

In terms of morphology (Fig. 7D), the depots at 25 °C were solid with a dense and smooth outer layer, while the depots at 45 °C swelled with heterogeneous shapes and a fragile structure. In the reference IVR condition, the depots tended to stick to the bottom of the glass vial and to flatten overtime. They were very flexible and less fragile than the depots at 45 °C.

DMSO content released from the depots was not determined during the PK study. However, the solvent release was monitored during the *in vitro* release tests and was compared to the API release at each time point (Fig. S5). In contrast to the previous data obtained with meloxicam and bupivacaine formulations, the DMSO release at 25 °C and 45 °C with the flow-through apparatus was similar and largely above to the one observed with the reference IVR setup. As previously observed, at 45 °C, the DMSO/API release ratio was around 1, highlighting a similar release kinetic for both components. For the 25 °C condition, the DMSO release was faster than the API release, with a decreasing ratio overtime. On the other hand, the DMSO release in the reference IVR setup tended to be slower than the API release, with a ratio below 1 from Day 1.

Finally, water uptake was measured at Day 4 for the different IVR conditions. This time, the highest water uptake was observed for the reference IVR setup (*i.e.* +328 % from initial depot weight) which was 2.1 and 1.4-fold higher than the water uptake measured for the flow through apparatus at 25 °C and 45 °C (+159 % and +242 % respectively). The water uptake was again higher at 45 °C than at 25 °C, as it was observed with meloxicam and bupivacaine formulations studies.

## 5 Discussion

The present article focused on improving the *in vivo* predictability of IVR profiles of BEPO® based formulations an *in situ* forming depot (ISFD) technology. Historically, translatability of the *in vitro* results has been challenging and API dependent, leading to cases with excellent IVIVC and to others with a lower predictability level. For this study, three model formulations, containing either meloxicam, bupivacaine or EFdA, were used. These formulations were initially tested *in vitro* using a reference IVR method, derivative of a sample and separate setup, using half of a gelatin capsule as a mold to obtain reproducible depot shapes. It was highlighted that none of them had an *in vitro* release profile translatable to *in vivo* data, mainly because of a poor simulation of the initial burst. In the case of the meloxicam and bupivacaine formulations, the API were selected as models as they present different physico-chemical properties. Meloxicam displays a negative charge at pH 7.4 because of a keto-enol tautomerization mechanism, and its solubility increases with increasing pH. On the other hand, bupivacaine is a base (pKa 8.2) that presents some positive charge (+0.75) at pH 7.4, and whose solubility increases with decreasing pH. These different physico-chemical properties could be linked to subtle differences in terms of depot behavior *in vitro* or *in vivo*. Moreover, according to the USP chart, these two API are classified as very slightly soluble molecules (in the range of 0.1 to 1 mg/mL). However, their calculated logD at pH 7.4 or the one reported in the literature is 0 and 2.6 for meloxicam and bupivacaine respectively, suggesting a greater solubility difference between them. In this case, meloxicam and bupivacaine formulations were injected subcutaneously in rats at the same volume and with the same polymeric composition. For bupivacaine formulation, after the burst phase (Day 1), the *in vitro* and *in vivo* release rates were similar. On the other hand, the release rate was slower for meloxicam formulation during testing with an increased discrepancy between *in vitro* and *in vivo* data after Day 1. This suggests that the reference IVR method does not generate the same predictability level for formulations of similar compositions and is highly dependent on the API tested. It is noteworthy that the formulation with meloxicam is more soluble in the testing conditions than the formulation with bupivacaine, which could suggest the current IVR model is more predictable for formulations using API of lower solubilities. In the case of the EFdA formulation, the 4′-Ethynyl-2-fluoro-2′-deoxyadenosine is more soluble that the previously tested API (>800 µg/mL) and the *in vitro* profiles obtained largely underestimated the *in vivo* PK profile after subcutaneous injection in dogs, with an even less reliable burst estimation than with the meloxicam and bupivacaine formulations.

Facing this variability in the translatability of IVR results, a custom-made dissolution apparatus based on the USP Apparatus 4 flow-through principle was developed, involving more biorelevant aspects. First of all, it is known that the release behaviour is highly dependent on sink conditions for some drugs (Hate, Thompson, and Singaraju 2024), and a large volume of medium will not be encountered at the injection site. Thus, a flow-through principle was favored, to have a minimal but also a regularly renewed release buffer, more representative of the injection site microenvironment and of the drug absorption dynamics. This was made possible through the Volume Flow Controller (VFC) module which allows a precise regulation of the buffer flow rates. In the presented experiments, the targeted flow rate varied of ±15 % at worst. Another aspect involves the drug product environment during testing. In classic USP release systems, formulations are injected directly into the release buffer which might not be appropriate to get biorelevant ISFD depot shapes and internal structures. One of the main challenges to tackle is thus to constrain the formulation during the full testing, as it would be after a parenteral injection. In an attempt to mimic a subcutaneous injection, it was decided to use a hydrogel matrix as a surrogate of the subcutaneous tissue, to constrain the formulation during the depot formation and the release phase. In this setup, it was essential to reduce the depot shape variability at injection (the agarose matrix acting here as a constraining matrix). Also, from a release perspective, it was previously demonstrated that *in situ* forming depots are sensitive to environmental tissues pressure, leading to variations in the drug release rate (Patel et al., 2010, Hernandez et al., 2016, Kempe and Mader, 2012). Containing the depot within a matrix at the formation stage and during the whole release duration is thus a relevant parameter to integrate during *in vitro* release testing. The new flow-through release system presented here intends to mimic the subcutaneous tissue with an agarose-based hydrogel matrix. Finally, the system was made so that the temperature and buffer flow can be controlled and tailored if needed, allowing a large range of conditions to tune for optimizing the *in vivo* predictability.

In a first set of experiments using meloxicam and bupivacaine formulations, *in vitro* data were compared to *in vivo* data obtained from the *ex vivo* dosage of depots formed after subcutaneous injection in rats. Depots from the model formulations were recovered from the animals at different time points, dissolved and the remaining API cargo was quantified, allowing the generation of an API release profile. *In vitro*, data were generated over the full study duration using a reference IVR method at 37 °C (Suh et al., 2021, Roberge et al., 2020). Different conditions were also investigated using the innovative flow-through apparatus, at temperatures of 25 °C and 45 °C as well as under flow rates ranging from 1 mL/h to 10 mL/h.

It was shown that changing the parameters of the flow-through apparatus allowed the API release kinetics to be tailored. In particular, temperature was a critical variable. The higher the temperature, the more pronounced the API release, especially the prominence of the burst release at Day 1. In contrast, variation of the flow rate had a minimal impact on API release for these model formulations. Unexpectedly, the release profiles at 45 °C were comparable to the *in vivo* releases for both meloxicam and bupivacaine formulations. On the other hand, the release profiles generated at 25 °C were closer to the ones obtained with the reference IVR setup at 37 °C. Overall, a temperature variation of ±4°C was observed for sets at 25 °C (*i.e.* left at room temperature), while the variation was only of ±1.5 °C for temperatures above 25 °C (*i.e.* controlled by the heating system of the flow-through apparatus). Further characterization was thus conducted to better understand the mechanisms governing the release.

API release from *in situ* forming depots is driven by multiple mechanisms, among them: i) the solvent release kinetics through the phase exchange phenomenon, ii) the drug diffusion through the polymer matrix (influenced by the API aqueous solubility, the depot surface area, the depot porosity…) and iii) the progressive matrix erosion at a later stage of the release (Parent et al., 2013, Wright and Burgess, 2012, Graham et al., 1999, Zhang and Fassihi, 2020). The depot morphology and internal structure are thus profoundly linked to the release performances (Kempe and Mader, 2012, Zhang and Fassihi, 2020).

*In vivo*, depots are formed in an organized tissue matrix (*i.e.* subcutaneous tissue), at temperatures approximating 34 °C (Kinnunen and Mrsny 2014), leading to depots of ellipsoidal shape (Peloso et al., 2021, Ng et al., 2023). For the tested BEPO® formulations, the solvent release is fast with more than 60 % released within the first 24 h, leading to a minimal water intake and subsequent depot swelling. A thick and rigid outer-layer is observed around the depots, with the presence of a thickening fibrotic capsule over time which is a classic inflammatory response to a foreign body (Anderson et al., 2008, Paquette et al., 2014, Ng et al., 2023). In terms of internal structure, depots generally display “finger-like” porous structures, characteristic of a rapid phase-inverting system (Parent et al., 2013, Roberge et al., 2020).

In the reference IVR setup, BEPO® depots precipitate initially within the gelatin capsule, adopting most often the ellipsoidal shape of this mold. At a macroscopic level, the depot outer-layer is solid with a “sponge-like” structure, characteristic of a slower phase-inversion system compared to *in vivo* (Ng et al. 2023). Drug release is also driven by the copolymer erosion in the *in vitro* release medium. Degradation studies carried out with the same polymeric ISFD technology (Ng et al. 2023) previously highlighted the different solvent exchange rates between *in vivo* and the reference *in vitro* setup, with a faster copolymer precipitation *in vivo* leading to the formation of smaller pores compared to a more porous depot *in vitro*. Moreover, these characteristics have shown an increased water influx and degree of swelling, especially with polymers of low molecular weight like those used in this study (Ng et al., 2023, Liu and Venkatraman, 2012, Bode et al., 2018, Patel et al., 2010).

In this flow-through IVR setup, the temperature greatly affected the depot precipitation and subsequent API release. At 25 °C, the depots precipitate at rates similar to *in vivo* depots (*i.e.* similar DMSO release rate), leading to rigid structures with a minimal swelling and water uptake. On the other hand, depots at 45 °C have a slower DMSO release, leading to fragile structures, with a large surface area and an important water uptake. These properties can also be associated with an increased copolymer chain mobility at temperatures above their glass transition temperature (Ng et al., 2023, Shafiee et al., 2024). The depot increased surface area could also be linked to a weaker agarose network at 45 °C and a less structured hydrogel to constrain the implant over the course of the study.

*In vivo*, it is suspected that the presence of a matrix and subsequent creation of a fibrotic capsule around the depot drives an important part of the API release from the depots, due to a compression of the polymeric structure. API absorption can also be promoted at later time points by the foreign body reaction in place, leading to a vascularization of the fibrotic capsule and the drastic chemical changes provoked in an attempt to destroy the foreign material (*e.g.* pH changes and macrophages recruitment) (Anderson, Rodriguez, and Chang 2008). *In vitro*, the API release seems mainly linked to speed of initial precipitation and the DMSO burst, later followed by passive diffusion through the porous matrix and erosion. Hence, the previously described *in vitro* setup may underestimate the initial API burst, leading to a lower IVIVR.

At this point, using an IVR setup with a hydrogel at a higher temperature and under a continuous buffer flow seems to improve the translatability of the initial API release from *in vitro* to *in vivo* data. The formation of a larger depot surface area and larger pores could be the main factor driving the API release in this setup at 45 °C, while the increase of the temperature could also promote a faster release through an increase of the API solubility in the release medium. This hypothesized release mechanism differs greatly from what is expected in an *in vivo* environment, meaning the addition of biorelevant parameters was not sufficient to copy the *in vivo* mechanisms. Instead, the release kinetics were comparable to the initial goal, even if deviating from *in vivo* conditions.

The flow-through apparatus was finally tested with a third formulation composition with human-relevant doses and compared to a classic PK study (*i.e.* no ADME bypass). It validated the likeliness to inject large volumes of *in situ* forming depot formulations (*i.e.* 600 µL) in the equipment without altering its flow control efficiency. Results were again conclusive as an improved IVIVR was observed when using the flow-through apparatus at 45 °C. Although the API burst extent was not accurately mimicked (*i.e.* lower *in vitro* release compared to *in vivo* at the 6 h time point), it was much better matched at Day 1. With this formulation, the result’s variability tended to be higher than with meloxicam and bupivacaine formulations and could be associated to the polymeric composition of the formulation (*i.e.* lower PEG lengths leading to a slower DMSO release, higher water influx (Patel et al. 2010) and potentially less reproducible microstructure), API nature (*i.e.* higher solubility largely promoted by the temperature increase) and/or larger injected volume (*i.e.* 580 µL vs 170 µL in the first tests). Moreover, the use of the flow-through IVR apparatus helped to improve the variability compared to the reference IVR method (Fig. S3 C.). Also, the mechanical properties of the depots were in line with those observed in the initial flow-through equipment testing, confirming the robustness of the technique.

This study allowed to improve the current reference IVR method used for BEPO® *in situ* forming depot technology, although it was not accomplished by fully mimicking the *in vivo* mechanisms as illustrated for instance by the DMSO/API release ratio and the large swelling of the depots incubated at 45 °C. Table 3 qualitatively compares the different outcomes observed in all the release setups.

**Table 3:** Qualitative comparison of the different *in vitro* setups tested. The reference frame is the *in vivo* data. Each outcome is compared as higher (+), lower (−) or similar (≡) compared to the *in vivo* data. Some parameters can be deemed even higher (+ +) or lower (− −) than the other *in vitro* setups.

Comparison to *in vivo* data	*In vitro* reference at 37 °C	*In vitro* flow-through at 25 °C	*In vitro* flow-through at 45 °C
Compression	− −	−	−
Water intake	+	+	+ +
Depot outer shell thickness	−	−	− −
DMSO release	−	−	−
API release	−	−	≡

These unexpected results will lead to further development of the flow-through IVR equipment. A larger screening should be conducted with more APIs of different solubilities to confirm the robustness of the equipment, as well as with other copolymers or polymeric technologies. However, using a high temperature for the release could be unfavourable for some unstable APIs as well as formulations with specific release mechanisms. Consequently, improvements on the equipment should be performed to keep the same API release profile with lower temperatures. Modification of the IVR setup should be made to try to be more biorelevant and mimic the *in vivo* release mechanisms as much as possible. The hydrogel should thus be optimized to induce higher constraint around the depot while still presenting an acceptable resistance to the flow rate. The buffer composition could also be tailored to improve the release from the depots, by changing its components, the ionic force or the pH. Finally, the addition of an UV analysis module is being installed to quantify the API released from each reactor channel. This will allow to further automatize the apparatus by quantifying the API released over short intervals of time, allowing a follow-up of the release in real-time.

## 6 Conclusion

In this study, an innovative dissolution apparatus was tested to optimize *in vivo* translatability of *in vitro* release profiles from *in situ* forming depots, using BEPO® technology as a model. Thanks to the hydrogel matrix, the depots were constrained at the formation stage, resulting in reproducible shapes at release study initiation. It was highlighted that the temperature was a critical parameter for API release from polymeric matrices, and that a high temperature (*i.e.* 45 °C) could lead to release kinetics comparable to *in vivo* profiles when using this IVR setup. As the mechanical properties were different between depots formed *in vivo* and *in vitro* in the flow-through apparatus at 45 °C, it is hypothesized that the new IVR system promotes API release from BEPO® formulations through different release mechanisms than those involved *in vivo*. However, these mechanisms still allowed an improvement of the *in vitro* results translatability to *in vivo*. Further work should be performed to assess if such an improvement can be generalized to polymeric phase-inverting *in situ* forming LAI.

From these initial tests, the current flow-through setup will continue to be tested with other formulation compositions and APIs to confirm the robustness of the system. In parallel, additional optimization of the equipment will be made to get closer to the *in vivo* release mechanics, by modification of the constraining hydrogel or buffer composition for example.

## CRediT authorship contribution statement

**Charlotte Peloso:** Writing – original draft, Visualization, Methodology, Investigation, Formal analysis, Conceptualization. **Etienne Yvorra:** Investigation. **Romain Delamare:** Writing – original draft, Software, Methodology, Conceptualization. **Mélanie Campana:** Writing – review & editing, Project administration. **Sylvestre Grizot:** Writing – review & editing, Project administration. **Adolfo Lopez-Noriega:** Writing – review & editing, Supervision, Methodology, Conceptualization.

## Declaration of competing interest

The authors declare the following financial interests/personal relationships which may be considered as potential competing interests: [Adolfo Lopez-Noriega reports financial support was provided by Bill and Melinda Gates Foundation. Adolfo Lopez-Noriega reports a relationship with MedinCell SA that includes: employment and equity or stocks. Charlotte Peloso reports a relationship with MedinCell SA that includes: employment and equity or stocks. Etienne Yvorra reports a relationship with MedinCell SA that includes: employment and equity or stocks. Romain Delamare reports a relationship with MedinCell SA that includes: employment and equity or stocks. Melanie Campana reports a relationship with MedinCell SA that includes: employment and equity or stocks. Sylvestre Grizot reports a relationship with MedinCell SA that includes: employment and equity or stocks. Romain Delamare has patent #PCT/EP2022/058434 pending to MedinCell SA. This publication is based on research funded in part by the Bill & Melinda Gates Foundation (OPP1213780). The findings and conclusions contained within are those of the authors and do not necessarily reflect positions or policies of the Bill & Melinda Gates Foundation. If there are other authors, they declare that they have no known competing financial interests or personal relationships that could have appeared to influence the work reported in this paper].

## Data Availability

The authors do not have permission to share data.
