# Supplementary material for: Improving in vivo release prediction from in situ forming depots with a novel flow-through in vitro dissolution apparatus
Source: Int J Pharm. 2025 Aug 20;681:125884. doi: 10.1016/j.ijpharm.2025.125884 (PMC12344484; doi:10.1016/j.ijpharm.2025.125884)
Supplement: Supplementary Data 1 [file mmc1.pdf]

Improving *in vivo* release prediction from *in situ* forming depots with a novel flow-through *in vitro* dissolution apparatus

Supplementary information

Charlotte Peloso[a], Etienne Yvorra[a], Romain Delamare[a], Mélanie Campana[a], Sylvestre Grizot[a] and Adolfo Lopez-Noriega[a]*

*Corresponding author: adolfo.lopeznoriega@medincell.com

[a] MedinCell S.A., 3 rue des frères Lumière, 34830 Jacou, France

**Compound to quantify**	**Meloxicam**	**Bupivacaine**	**EFdA**	**DMSO**
**Equipment**	ACQUITY UPLC H-Class PLUS			1260 Infinity II LC
**Column**	Acquity UPLC BEH C18 130 Å 1.7 µm		Acquity UPLC HSS T3 100 Å 1.8 µm	Infinity Poroshell 120 EC-C18 4 µm
**Column temperature**	30°C		38°C	25°C
**Organic eluent**	ACN:TFA (100:0.1 v/v)		ACN:FA (100:0.1 v/v)	ACN
**Aqueous eluent**	H_2O:TFA (100:0.1 v/v)		H_2O:FA (100:0.1 v/v)	H_2O
**Flow rate**	0.30 mL/min	0.61 mL/min	0.30 mL/min	0.90 mL/min
**Detection**	UV at 352 nm	UV at 210 nm	UV at 260 nm	UV at 210 nm

Table S 1: Liquid chromatography operating conditions for dosage of the different compounds. ACN: Acetonitrile, TFA: Trifluoroacetic acid, FA: Formic Acid.

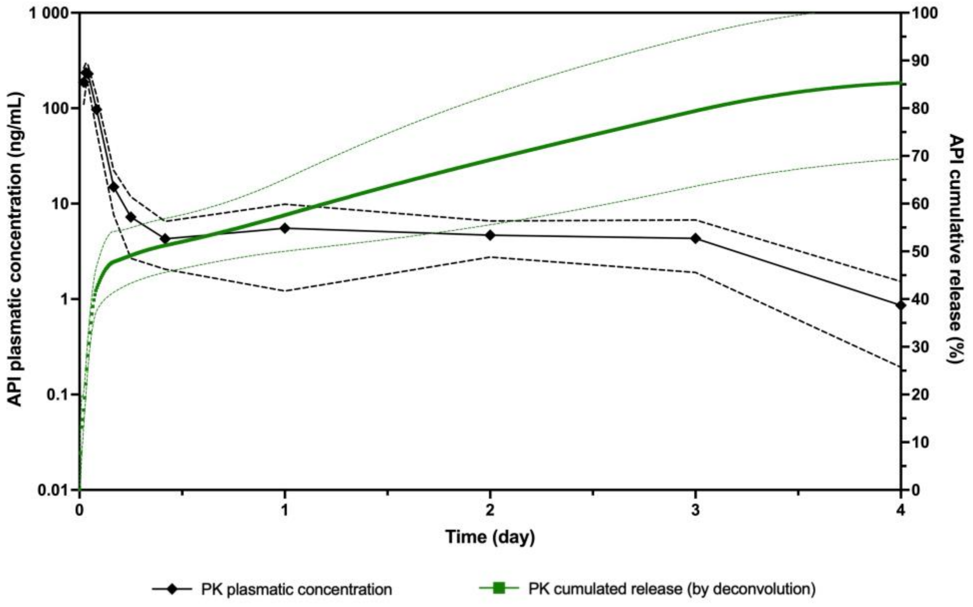

Figure S 1: Conversion of PK data from the EFdA formulation to cumulative in vivo release. The API plasmatic concentration measured in vivo is represented in log/lin on the left axis while the corresponding cumulative in vivo release percentage is plotted in lin/lin on the right axis. For the plasmatic concentrations, the lower limit of quantification was of 0.01 ng/mL. Data are presented as mean values in full lines and standard deviation in dotted lines.

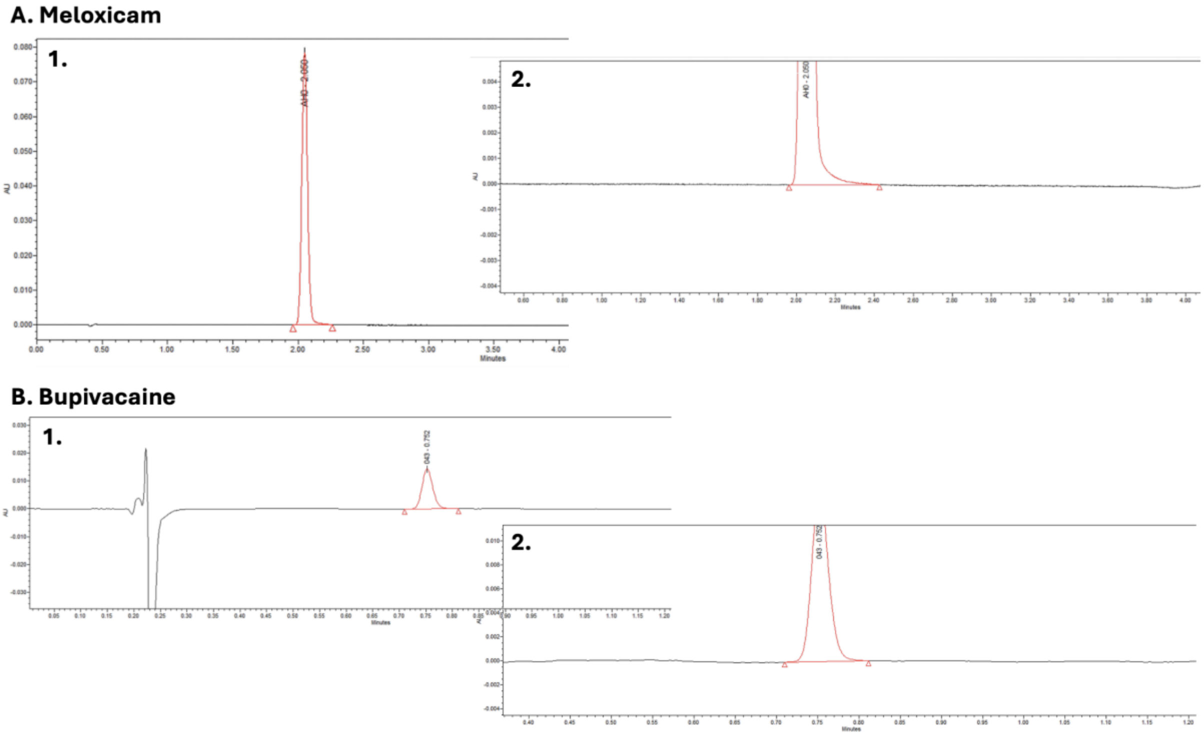

Figure S 2: Chromatograms of meloxicam (A) and bupivacaine (B) recovered after 15 days at 45°C in PBS. 1. Full chromatograms. 2. Zoomed chromatograms on the baseline to identify potential impurities.

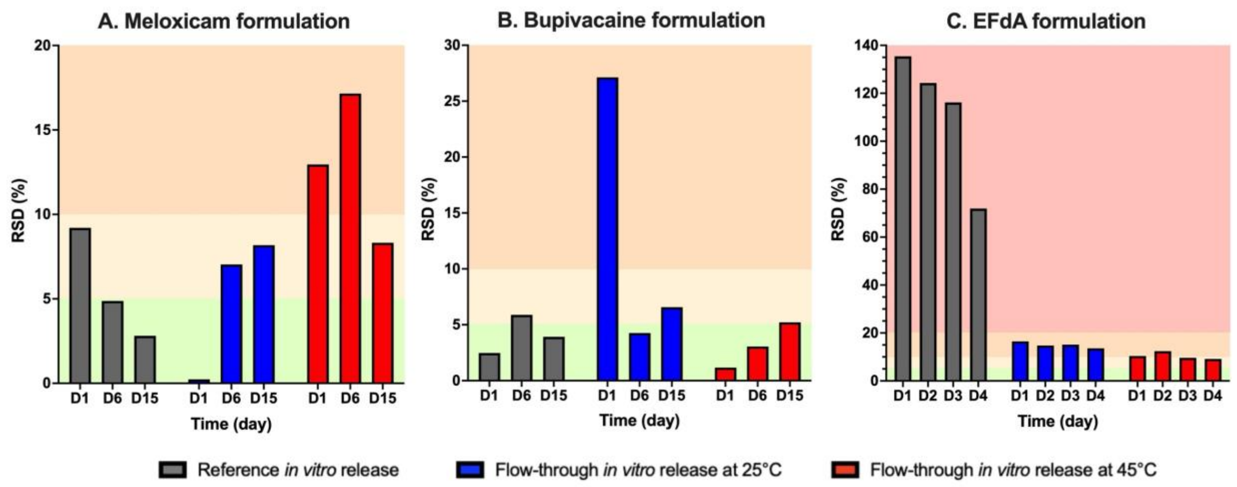

Figure S 3: Comparison of Relative Standard Deviations (RSD) of meloxicam (A), bupivacaine (B) and EFdA (C) formulations for each release setup. Flow-through rate was at 1 mL/h for meloxicam and bupivacaine formulations and 5 mL/h for EFdA formulation. RSD is considered: <5% (in green) acceptable for quality control; <10% (in yellow) acceptable for development activities; < 20% (in orange) acceptable for research activities; >20% (in red) not acceptable.

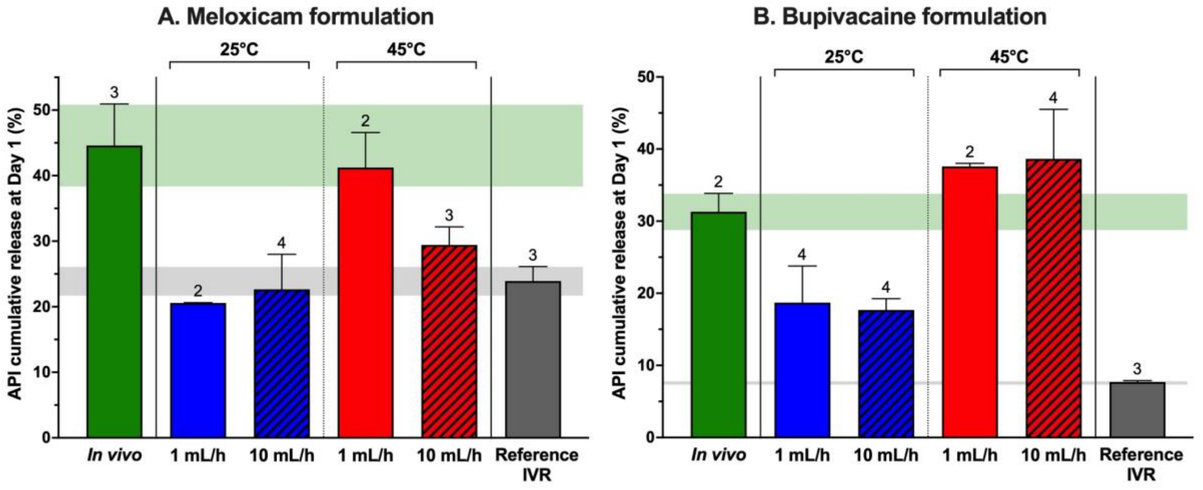

Figure S 4: Comparison of API releases from meloxicam (A) and bupivacaine (B) formulations at Day 1 in all flow-through IVR conditions.

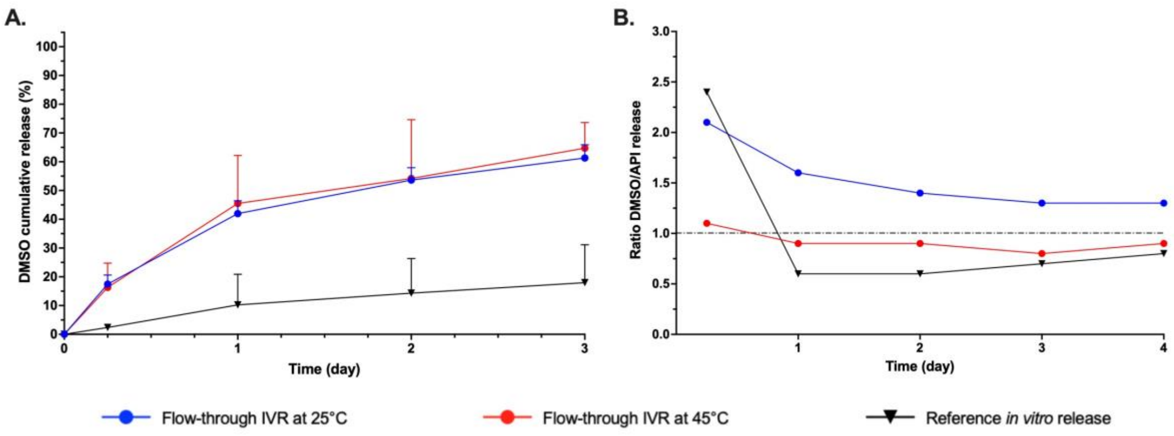

Figure S 5: Comparison of the DMSO release from EFdA formulation. A. DMSO cumulative percentage releases in different in vitro setups. B. Ratio of DMSO/API percentage release during the study.
